# Current axillary management of patients with early breast cancer and low-volume nodal disease undergoing primary surgery: results of a United Kingdom national practice survey

**DOI:** 10.1007/s10549-024-07328-4

**Published:** 2024-05-10

**Authors:** K. Fairhurst, S. A. McIntosh, R. I. Cutress, S. Potter, Our Citable Collaborators, Nick Abbott, Mohammad Abdullah, Avi Agrawal, Laura Arthur, Amina Bouhelal, Rachel Bright-Thomas, Victoria Brown, Sundas Butt, Charlie Chan, Wilson Cheah, Adamantia Chrysafi, Sarah Clark, Ramsey Cutress, Urszula Donigiewicz, Hannah Dunlop, Donna Egbeare, Waleed Fahmy, Douglas Ferguson, Nina Gill, Daniel Glassman, Tomasz Graja, Kelvin Francis Gomez, Amit Goyal, Ahmed Hamad, Anita Hargreaves, Grant Harris, Thomas Hubbard, Alex Humphreys, Javeria Iqbal, Eliana Kalakouti, Charlotte Kallaway, Isabella Karat, Sabeeha Karim, Saira Khawaja, Polly King, Eleftheria Kleidi, Hannah Knight, Jaroslaw Krupa, Alexander Leeper, Valentina Lefemine, Rebecca Lewis, Richard Linforth, Emma MacInnes, Mhairi Mactier, Christina Mamareli, Yazan Masannat, Tahir Masudi, Ross McLean, Rob Milligan, Bijan Ansari Mohabadian, Jenna Morgan, Asma Munir, Claire Murphy, Soudamini Nayak, Keith Ogston, Robert Price, Mujahid Pervaz, Saed Ramzi, Tim Rattay, Azel Regan, Laszlo Romics, Claire Louise Rutherford, Sunita Saha, Ibrahim Sallam, Subodh Seth, Lisa Sheehan, Amanda Thorne, Raghavan Vidya, Kate Williams, Mina Youssef, Shaista Zafar

**Affiliations:** 1 Centre for Surgical Research, Department of Population Health Sciences, Bristol Medical School, University of Bristol, Bristol, UK; 2 Patrick G Johnston Centre for Cancer Research, Queens University Belfast, 97 Lisburn Road, Belfast, BT9 7AE Northern Ireland; 3 Somers Cancer Research Building, University of Southampton and University Hospital Southampton, Tremona Road, Southampton, SO16 6YD UK; 4 Centre for Surgical Research, Department of Translational Health Sciences, Bristol Medical School, University of Bristol, Bristol, UK

**Keywords:** Targeted axillary dissection, Axillary node clearance, Breast cancer, Low volume nodal disease, Trial feasibility

## Abstract

**Purpose:**

UK NICE guidelines recommend axillary node clearance (ANC) should be performed in all patients with biopsy-proven node-positive breast cancer having primary surgery. There is, however, increasing evidence such extensive surgery may not always be necessary. Targeted axillary dissection (TAD) may be an effective alternative in patients with low-volume nodal disease who are clinically node negative (cN0) but have abnormal nodes detected radiologically. This survey aimed to explore current management of this group to inform feasibility of a future trial.

**Methods:**

An online survey was developed to explore current UK management of patients with low-volume axillary disease and attitudes to a future trial. The survey was distributed via breast surgery professional associations and social media from September to November 2022. One survey was completed per unit and simple descriptive statistics used to summarise the results.

**Results:**

51 UK breast units completed the survey of whom 78.5% (*n* = 40) reported performing ANC for all patients with biopsy-proven axillary nodal disease having primary surgery. Only 15.7% of units currently performed TAD either routinely (*n* = 6, 11.8%) or selectively (*n* = 2, 3.9%). There was significant uncertainty (83.7%, *n* = 36/43) about the optimal surgical management of these patients. Two-thirds (*n* = 27/42) of units felt an RCT comparing TAD and ANC would be feasible.

**Conclusions:**

ANC remains standard of care for patients with low-volume node-positive breast cancer having primary surgery in the UK, but considerable uncertainty exists regarding optimal management of this group. This survey suggests an RCT comparing the outcomes of TAD and ANC may be feasible.

**Supplementary Information:**

The online version contains supplementary material available at 10.1007/s10549-024-07328-4.

## Background

Breast cancer affects almost 56,000 women every year in the United Kingdom (UK) [1], the majority of whom will have surgery as their first treatment. As part of their diagnostic assessment, all UK patients will have an ultrasound scan (USS) of their axilla with biopsy of any suspicious/indeterminate nodes to establish whether the cancer has spread and inform the type of axillary surgery that is performed. Current National Institute of Health and Care Excellence (NICE) guidance states that all women with biopsy-proven node-positive breast cancer should be offered an axillary node clearance (ANC), irrespective of the extent of disease [2].

ANC is a highly morbid procedure with one in three women experiencing significant, lifelong complications including lymphoedema and chronic pain that can dramatically impact their quality of life [3, 4]. Furthermore, there is no evidence that this extensive surgery improves breast cancer survival [5–13]. Internationally, the use of ANC is declining [14] based on the results of the landmark Z0011 study which showed that ANC did not improve survival in patients with clinically node-negative (cN0) disease found to have 1–2 involved nodes on sentinel node biopsy (SNB) [5–9] in women with T1 or T2 invasive primary breast cancer. In the UK, exploring alternatives to ANC in patients with node-positive breast cancer was identified as the top research priority in breast cancer surgery in a recent James Lind Alliance Priority Setting Partnership [15].

Targeted axillary dissection (TAD) is a new procedure that may provide an effective alternative to ANC in patients with low-volume nodal disease—i.e. those who are cN0 but have 1–2 involved nodes detected on USS. TAD combines a sentinel node biopsy with targeted removal of the involved node(s) that are localised prior to surgery. Use of TAD addresses concerns about high false-negative rates with SNB alone in patients with nodal involvement [16], facilitating removal of known disease, and providing accurate staging information to guide adjuvant treatment decision making whilst allowing patients to avoid the morbidity associated with an ANC. The technique has been shown to be feasible [17] and is now standard of care in node-positive patients who have a complete response to neoadjuvant chemotherapy [18]. There are also emerging data to support the use of TAD in patients having primary surgery [19].

However, there is a need to robustly evaluate TAD in the primary surgical setting before it is introduced into routine clinical practice. Ideally a randomised clinical trial (RCT) is needed but it is important that a future trial is well designed, reflects UK practice and addresses a question that is important to both clinicians and patients. This survey aims to explore the current management of patients with low-volume axillary nodal disease having primary surgery in the UK to inform the feasibility, design and conduct of a future RCT comparing surgical techniques.

## Methods

An online national practice survey was developed in SurveyMonkey®. Questions explored the current management of patients with early breast cancer who have biopsy-proven low-volume axillary nodal disease defined as clinically node negative (cN0—on physical examination only) with no more than two suspicious nodes on axillary USS. Further questions focused on the feasibility and design of a future RCT comparing TAD and ANC in the primary surgery setting. (Appendix 1: National Practice Survey).

All breast surgery units in the UK were invited to complete the survey on behalf of their multidisciplinary teams (MDTs). The survey was distributed via social media and the UK breast surgery professional associations [Association of Breast Surgery (ABS) and The Mammary Fold, (UK trainee breast surgery group)] between September and November 2022. Regular reminders were sent via email and association newsletter to maximise participation. Simple descriptive statistics were used to summarise results.

## Results

Surveys were completed by 51 UK breast units (of 130 UK units [20], 39%), most of whom treated between 201 and 600 new breast cancers per year (*n* = 36, 70%) (Table 1). For most centres, this included between 25 and 50 patients with low-volume axillary nodal disease annually (Table 1).

**Table 1:** UK breast unit demographics

Question	Proportions
Hospital region (*n* = 51 units)	*n*
Southwest	10
Scotland	6
Yorkshire and the Humber	5
West Midlands	5
Southeast	4
London	4
East Midlands	3
Wales	3
East of England	3
Northwest	2
Northeast	2
Northern Ireland	1
Republic of Ireland	1
Approximate number of breast cancers treated/year (*n* = 51 units)	*n* (%)
< 200	3 (6)
201–400	21 (41)
401–600	15 (29)
601–800	6 (12)
> 801	6 (12)
Number of clinically node negative, radiologically detected low-volume lymph nodal disease at early diagnosis of breast cancer/year (*n* = 49 units)	*n* (%)
< 25	10 (20.5)
25–50	22 (45)
51–100	11 (22.5)
> 100	6 (12)
Missing responses	2

The majority of units (*n* = 40, 78.5%) reported that they would perform an ANC for patients with low-volume nodal disease having primary surgery. A minority of units (*n* = 8, 15.7%) reported performing TAD, either routinely (*n* = 6, 11.8%) or in certain circumstances (*n* = 2, 3.9%) and a few (*n* = 3, 5.8%) performed SNB in this context. Almost three quarters (38/51, 74.5%) of units were performing TAD in the neoadjuvant setting. Most units (*n* = 31/38, 81.6%) reported that a TAD comprised a dual tracer SNB combined with removal of pre-operatively localised node(s), although variations in the technique were highlighted (Table 2).

**Table 2:** Current management of patients with low-volume axillary nodal disease in the UK

Question	Proportions *n* (%)
Type of axillary surgery performed in cN0 radiologically detected low-volume nodal disease in the primary surgery setting (*n* = 51 units)	
ANC	40 (78.5)
TAD	6 (11.8)
SLNB	3 (5.8)
TAD or ANC depending on age and tumour grade	2 (3.9)
Situations when TAD is performed (*n* = 46 responses from 38 units, *n* = 9 dual response; total not 100%)	
Post NACT convert from LN + to LN- within RCT (e.g. ATNEC)	17 (44.7)
Post NACT convert from LN + to LN- as standard of care	17 (44.7)
Selected low-volume LN + disease converting to LN- post NACT	7 (18.4)
Selected low-volume LN + having primary surgery	3 (7.9)
For all with low-volume LN + disease having primary surgery	2 5.2)
TAD technique (*n* = 38 units)	
Dual tracer SLNB and removal of pre-operatively localised nodes	31 (81.6)
Dual tracer SLNB and removal of palpably abnormal nodes	3 (7.9)
Single tracer SLNB (dual only if fails) and removal of preop localised nodes	3 (7.9)
Removal of localised abnormal nodes only	1 (2.6)

*USS* = *cN0* clinically lymph node negative, *ANC* axillary node clearance, targeted axillary dissection, *SLNB* sentinel lymph node biopsy, *NACT* neo-adjuvant chemotherapy, *LN* lymph node

The majority of participating units (*n* = 36/43, 83.7%) reported uncertainty about the optimal surgical management of patients with low-volume nodal disease undergoing primary surgery and two thirds of MDTs (*n* = 27/42, 64.3%) felt that a future trial comparing TAD and ANC may be feasible. Most respondents felt that the trial should include both pre and post-menopausal patients with all molecular subtypes of breast cancer who had 1–2 involved nodes on USS (Table 3). Locoregional recurrence (LRR) was considered the most important primary outcome for a future trial by clinicians completing the survey.

**Table 3:** Feasibility of a UK trial comparing TAD to ANC for clinically node negative, radiologically detected low-volume nodal disease at diagnosis of early breast cancer

Question	Proportions *n* (%)
How does your MDT feel about the surgical management of cN0 patients with low-volume, radiologically detected nodal disease at diagnosis (*n* = 43 units)	
Uncertainty about best surgical management—RCT needed	36 (83.7)
No uncertainty—TAD is best treatment	4 (9.3)
No uncertainty—ANC is best treatment	2 (4.7)
Uncertainty about whether an RCT is needed	1 (2.3)
Missing responses	8
Does your MDT feel a trial of TAD versus ANC for cN0, low-volume radiologically detected nodal disease at diagnosis is feasible? (*n* = 42 units)	
Yes	27 (64.3)
Not sure	11 (26.2)
No	4 (9.5)
Missing responses	9
Would your MDT be willing to recruit to a future trial comparing TAD and ANC in cN0, low-volume radiologically detected nodal disease at diagnosis who are having primary surgery? (*n* = 41 units)	
Yes	29 (70.7)
Unsure	9 (22.0)
No	3 (7.3)
Missing responses	10
Would your Unit be interested in participating in a future trial? (*n* = 41 units)	
Yes	31 (75.6)
Unsure	9 (22.0)
No	1 (2.4)
Missing responses	10
For an RCT comparing TAD versus ANC:	
How many abnormal/indeterminate nodes on USS should be permitted for patients to be eligible for inclusion?	*n* = 40 units
1 node	3 (7.5)
1–2 nodes	25 (62.5)
Up to 3 nodes	8 (20.0)
Not happy to make assessment on USS	4 (10.0)
Missing responses	11
What molecular subtypes of breast cancer would your MDT be willing to recruit?	*n* = 41 units
All molecular subtypes	29 (70.7)
ER + Her2−	9 (22.0)
Other	3 (7.3)
Missing responses	13
What age groups would your MDT be willing to recruit?	*n* = 41 units
Pre- and post-menopausal	28 (68.3)
Post-menopausal only	9 (22.0)
Unsure	4 (9.7)
Missing responses	10
What would the MDT consider to be the most meaningful primary outcome?	*n* = 40 units
Locoregional recurrence	29 (72.5)
Combination of Locoregional recurrence, Lymphoedema, QoL, distant or invasive-DFS	3 (7.5)
QoL	3 (7.5)
Arm/shoulder function	1 (2.5)
Lymphoedema rates	1 (2.5)
Lymphoedema rates & Arm/shoulder function	1 (2.5)
OS & DFS	1 (2.5)
Distant recurrence	1 (2.5)
Missing responses	11

*MDT* Multi-Disciplinary Team, *cN0* clinically lymph node negative, *RCT* Randomised Controlled Trial, *TAD* Targeted axillary dissection, *ANC* Axillary node clearance, *USS* ultrasound scan, *ER* oestrogen receptor, *HER2* human epidermal growth factor receptor 2, *QoL* Quality of life, *DFS* Disease free survival, *OS* Overall survival

## Discussion

This survey suggests that ANC remains the standard of care for patients with low-volume nodal disease having primary surgery in the UK, in line with current NICE guidelines. Only a minority of units are currently offering TAD in this group, either routinely or in selected cases. There is, however, considerable uncertainty regarding optimal surgical management of these patients. A trial comparing TAD vs ANC is, therefore, necessary and likely to be feasible in the UK.

A number of previous studies including ACOSOG-Z0011 [5–9] and IBSCG 23-01 [10, 11], and several recent meta-analyses [12, 13], have shown no oncological benefit of ANC for cN0 patients with 1–2 involved nodes detected at sentinel node biopsy. These findings have been adopted into North American NCCN breast cancer guidelines [21] and have led to a decrease in the practice of ANC worldwide [22]. A number of confirmatory studies are ongoing including the UKANZ POSNOC [23] study, the results of which are awaited. Although patients in these studies fulfil the criteria for having ‘low volume nodal disease’; none of the studies aimed to address the optimal management of patients with biopsy-proven axillary nodal involvement at diagnosis. Node positivity in these studies was detected following surgical axillary staging (SNB) so these patients would be likely to have a lower burden of disease. A study specifically focusing on the management of patients with known node-positive disease at diagnosis comparing targeted removal of the abnormal nodes versus an ANC (the current standard of care), is needed to determine whether de-escalation of axillary surgery in patients with low-volume nodal disease is safe and effective.

TAD has been shown to be feasible [17] and more accurate in identifying and removing involved nodes than SNB alone [24, 25]. If TAD can safely and effectively replace ANC for patients with low-volume nodal disease having primary surgery, it would reduce patient morbidity, whilst continuing to provide accurate staging information which is important for prognostication and to inform appropriate selection of adjuvant therapies. TAD is already being used in the neoadjuvant setting in many units. UK Surgeons, therefore, have the necessary experience to be able to perform the procedure in the primary surgical setting in a future trial. The variation in TAD techniques identified by the survey, however, highlights the need for careful surgical quality assurance within the trial to agree the prohibited and mandatory components of the technique so that TAD can be standardised and delivered in consistent way across participating centres within the study [26].

Other studies evaluating TAD for node-positive patients having primary surgery are ongoing and include TADEN (NCT04671511); a Canadian prospective cohort study assessing the technical feasibility of TAD and the international TAXIS RCT (NCT03513614) [27]. TAXIS is comparing tailored axillary surgery (TAS) in combination with axillary radiotherapy with ANC in node-positive patients who have residual disease after neoadjuvant chemotherapy in addition to those having primary surgery. In TAXIS, all patients receiving more limited surgery will also receive nodal radiotherapy. As a future UK trial would aim to only include patients with low-volume nodal disease, the addition of radiotherapy is likely to represent overtreatment in this group so a trial specifically comparing the outcomes of TAD alone vs ANC is needed in the UK to change practice.

This national practice survey has informed the feasibility and design of a future axillary de-escalation study, but it has limitations. First, it only includes the views of a third of UK breast units so may not be representative of the views of the breast cancer community as a whole. Responses were, however, received from units of various sizes across the UK, the majority of whom felt that there was uncertainty regarding management of patients with low-volume nodal disease having primary surgery. Furthermore, over 30 units expressed an interest in participating in a future trial suggesting that effective recruitment to a future trial would be feasible. It is possible that unit practices may differ from those reported in the survey or that the survey reflected the views of the completing clinician, rather than the MDT as a whole. Centres may also have overestimated the numbers of potentially eligible patients that they see every year, but numbers are broadly consistent with known rates of nodal positivity at presentation, suggesting this is unlikely. This survey has, therefore, provided valuable information regarding the enthusiasm for and design of a future trial comparing TAD and ANC in the UK.

TAD may be a safe and effective alternative to ANC in patients with low-volume axillary nodal disease having primary surgery, but robust evaluation is necessary to prevent haphazard adoption of TAD and potential patient harm. UK practice is evolving rapidly as 15% of units surveyed have already adopted TAD in this setting, despite the absence of high-quality evidence to support a change in practice. De-escalation of treatment in the absence of robust confirmatory evidence risks compromising the significant progress made in effective treatments to improve breast cancer outcomes as a consequence of research over the last few decades. This survey suggests that a future RCT comparing TAD and ANC in the UK may be feasible and has provided insights into key elements of trial design including potential inclusion criteria and outcome selection, in addition to highlighting the need for surgical quality assurance to standardise the technique. In the process of designing any trial, comprehensive patient and public involvement is also essential to ensure that the trial evaluates outcomes of importance to patients as well as professionals. This will optimise the trial success and allow the top UK research priority in breast cancer surgery to be effectively addressed.

As a result of the feasibility data reported here and further extensive patient and public involvement work with Independent Cancer Patients Voice (ICPV) [28], TADPOLE: A multicentre, pragmatic, phase III randomised controlled trial comparing **T**argeted **A**xillary **D**issection vs axillary node clearance in patients with **PO**sitive axillary **L**ymph nodes in **E**arly breast cancer, has been designed, and is currently at the final stages of consideration for funding by the National Institute for Health and Care Research (NIHR) Health Technology Assessment (HTA) programme and will open to recruitment in 2025.

### Supplementary Information

Below is the link to the electronic supplementary material.

Supplementary file1 (PDF 179 kb)

## Data Availability

The datasets generated and analysed during this study are stored under the provisions of the National Data Protection Act and the University of Bristol requirements. Data may be made available to bona fida researchers only, on reasonable request to the corresponding author, after their host institution has signed a Data Access Agreement.

## Declarations

### Previous communication

Abstract (Poster at Association of Breast Surgery Conference): Fairhurst, Katherine et al*. Targeted axillary dissection in low volume nodal disease at diagnosis: UK practice survey (TADPOLE),* European Journal of Surgical Oncology, Volume 45, Issue 9, e249.
