# Supplementary material for: Current axillary management of patients with early breast cancer and low-volume nodal disease undergoing primary surgery: results of a United Kingdom national practice survey
Source: Breast Cancer Res Treat. 2024 May 10;206(3):465–71. doi: 10.1007/s10549-024-07328-4 (PMC11208217; doi:10.1007/s10549-024-07328-4)
Supplement: Supplementary file 1 — Supplementary file1 (PDF 179 kb) [file 10549_2024_7328_MOESM1_ESM.pdf]

Current practice of the management of low-volume radiologically-detected positive axillary lymph nodes in patients with early breast cancer

The TADPOLE MDT Survey

We are interested in understanding the *current surgical management* of patients with early breast cancer who are *clinically node negative* (no evidence of axillary disease on examination) but who have *low volume radiologically detected axillary nodal disease at diagnosis* (confirmed by FNA or core biopsy). This will inform the design of a *future trial* comparing *targeted axillary dissection (TAD) vs axillary node clearance (ANC)* in patients with a low burden of axillary nodal disease at diagnosis *having primary surgery* (surgery as their first breast cancer treatment, not patients receiving neoadjuvant therapy).

We would be extremely grateful if you could complete this brief survey to reflect how these patients are currently managed in your Unit.

Please complete 1 survey per Unit on behalf of your multidisciplinary team

Anyone completing the survey will be a ***named PUBMED citable collaborator***. If you are a ***trainee*** completing the survey on behalf of your Unit, ***please identify a supervising consultant who will also become a PUBMED citable collaborator***.

Thank in in advance of your participation
Kit Fairhurst & Shelley Potter on behalf of the TADPOLE study team

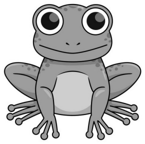

Current practice of the management of low-volume radiologically-detected positive axillary lymph nodes in patients with early breast cancer

Section 1 - Unit Details

1. Name of person completing survey

2. E-mail address of person completing survey

3. Name of Consultant (If trainee completing survey)

* 4. Name of Unit

5. Region

- ○ Scotland
- ○ Northern Ireland
- ○ Republic of Ireland
- ○ Wales
- ○ North East
- ○ North West
- ○ Yorkshire and Humber
- ○ East Midlands
- ○ West Midlands
- ○ South West
- ○ South East
- ○ London
- ○ East of England

6. Approximate ***number of breast cancers*** treated in your Unit ***per year***

- ○ <200
- ○ 201-400
- ○ 401-600
- ○ 601-800
- ○ >801

Current practice of the management of low-volume radiologically-detected positive axillary lymph nodes in patients with early breast cancer

Section 2 - Current management of patients with radiologically-detected nodal disease at diagnosis

7. Approximately how many patients who are ***clinically node negative,*** but are found to have ***radiologically-detected nodal disease*** at diagnosis does your Unit see ***each year***?

- <25
- 25-50
- 51-100
- >100

8. When ***assessing the axilla***, do the ***Radiologists*** in your Unit ***routinely quantify the extent of nodal disease*** seen on axillary USS?

- ***Yes*** - the absolute number of abnormal/indeterminate nodes seen on axillary USS is routinely reported in all patients
- ***Partially*** – reports only specifically state the number if one or two abnormal/indeterminate nodes are seen but use the term ‘multiple’ to classify more extensive nodal involvement
- ***No*** – reports only include whether or not abnormal/indeterminate axillary nodes are seen without further quantification
- ***Varies*** according to Radiologist performing the assessment

9. If they are having ***primary surgery***, what ***procedure*** do patients with ***low volume radiologically-detected nodal disease at diagnosis*** currently receive routinely in your Unit?

- Axillary clearance
- Sentinel node biopsy
- Targeted axillary dissection (defined as removal of involved node(s) + SNB)
- Other (please specify)

10. Do ***surgeons*** in your Unit currently ***perform targeted axillary dissection (TAD)***? (please tick all that apply)

- [ ] ***Yes*** – for ***all post-neoadjuvant*** patients who convert from node-positive to radiologically node-negative following treatment ***as standard of care***
- [ ] ***Yes*** – for ***post-neoadjuvant*** patients who convert from node positive to radiologically node-negative following treatment ***as part of a research study (e.g. ATNEC) only***
- [ ] ***Yes*** – for ***all*** women with ***low volume nodal disease*** having ***primary surgery***
- [ ] ***No*** – we are not currently performing TAD in any patients
- [ ] Yes – in certain circumstances – please provide details below

11. If surgeons in your Unit are performing ***targeted axillary dissection, how is this performed***?

- ○ Dual tracer sentinel node biopsy (SNB) + pre-operative localisation of abnormal or previously identified/clipped node(s)
- ○ Dual tracer SNB and removal of palpably abnormal nodes at the point of surgery (no pre-operative localisation of node(s))
- ○ Removal of localised abnormal/clipped/previously localised nodes only
- ○ Other (please provide details)

12. ***If clipped/abnormal nodes are localised*** pre-operatively, ***how is the localisation performed?***

- ○ Wire to clipped/abnormal node(s)
- ○ Magseed inserted in clipped/abnormal node(s)
- ○ Localizer inserted into clipped/abnormal nodes(s)
- ○ Saviscout to clipped/abnormal node
- ○ Tattooing
- ○ Varies by surgeon
- ○ Other method of localisation (please give details)

13. Is your Unit currently ***participating in*** or planning to participate in, the ***ATNEC study***?

- ◯ Yes – already participating in ATNEC
- ◯ Planning to participate/in set up
- ◯ No plans to participate in ATNEC

14. If your Unit is/is planning to participate in the ATNEC study, have they ***received training/mentorship in targeted axillary dissection***?

- ◯ Yes
- ◯ No
- ◯ Will have training/mentorship prior to study participation
- ◯ Already routinely perform TAD as part of their standard practice and do not require additional training/mentorship

Current practice of the management of low-volume radiologically-detected positive axillary lymph nodes in patients with early breast cancer

Section 3 - Feasibility of a future RCT

FEASIBILITY OF A FUTURE RCT COMPARING TARGETED AXILLARY DISSECTION AND AXILLARY CLEARANCE IN CLINICALLY NODE NEGATIVE PATIENTS WITH LOW VOLUME RADIOLOGICALLY DETECTED NODAL DISEASE AT DIAGNOSIS

A study to determine whether it is possible to ***avoid axillary clearance in patients with positive lymph nodes*** was identified as the ***top research priority*** to patients and professionals in the recent James Lind Alliance Priority Setting Partnership in breast cancer surgery.

We are therefore planning an ***RCT comparing targeted axillary dissection with standard axillary clearance*** in patients with ***early breast cancer who have a low burden of axillary nodal disease at diagnosis***. These will be the group of N1 patients who are ***clinically node negative but have radiologically-detected nodal disease confirmed on core biopsy/FNA***.

For the planned trial, a ***targeted axillary dissection would be defined as removal of pre-operatively localised involved node(s) in combination with a dual tracer sentinel node biopsy***.

We are interested in your Unit’s views about the feasibility and possible design of a future trial.

15. How does your MDT feel about the ***surgical management*** of clinically node negative patients with low volume, radiologically-detected nodal disease at diagnosis:

- ◯ There ***is uncertainty about the best surgical management*** of these patients (i.e. whether an ANC is necessary) and a trial is needed
- ◯ There is ***no uncertainty*** regarding the best surgical management of these patients and an ***ANC is the best treatment***
- ◯ There is ***no uncertainty*** regarding the best surgical management of these patients and a ***TAD is the best treatment***
- ◯ Uncertain about whether a trial is needed

16. Does your MDT feel that a ***trial comparing TAD and ANC*** in clinically node negative patients with low volume radiologically-detected nodal disease ***is feasible***?

◯ Yes ◯ No ◯ Unsure

17. Would your MDT recommend any ***additional axillary treatment after a targeted axillary dissection?***

- Yes
- No
- In certain situations (please provide details)

18. ***If yes***, what ***additional axillary treatment (if any)*** would your MDT recommend?

- Axillary radiotherapy above surgical dissection
- Axillary radiotherapy above surgical dissection +/- internal mammary chain (IMC) radiotherapy
- Other (please specify)

Inclusion/exclusion criteria for a future RCT

19. We would like to recruit patients with ***a low burden of axillary disease at diagnosis to a future trial***. ***How many abnormal/indeterminate nodes on USS should be permitted*** for patients to be eligible for inclusion in an ***RCT comparing TAD and ANC***?

- 1 node only
- 1-2 nodes
- Up to 3 nodes
- Would not be happy to make assessment based on axillary USS
- Other (please provide details)

20. What ***age groups*** would your MDT be willing to recruit to a ***trial comparing TAD and ANC?***

- Both pre and postmenopausal
- Postmenopausal only
- Unsure

21. What ***molecular subtypes*** of breast cancer would your MDT be willing to recruit to a ***trial comparing ANC and TAD*** in patients with radiologically detected nodal disease having primary surgery?

- ○ All molecular subtypes having primary surgery
- ○ ER positive HER-2 negative only
- ○ Other (please give details)

22. What would the MDT consider to be the ***most meaningful primary outcome*** for an ***RCT comparing TAD and ANC***?

- ○ Locoregional recurrence
- ○ Lymphoedema rates
- ○ Arm/shoulder function
- ○ Post-operative complications
- ○ Quality of life
- ○ Other (please give details)

23. Would your MDT be willing to ***recruit to a future RCT (TADPOLE) comparing TAD and ANC in clinically node negative patients with low volume radiologically detected nodal disease*** at diagnosis who are having ***primary surgery***?

○ Yes ○ No ○ Unsure

24. Would your Unit be interested in ***participating in a future trial***?

○ Yes ○ No ○ Unsure

If yes, please provide details for the person who would be the ***local PI*** for the study

25. PI Name

26. PI contact e-mail address

Thank you very much for completing the survey - your MDT's views will be important in helping to design the future trial
